# Perioperative nutritional risk and its influencing factors in patients with oral cancer: a longitudinal study

**DOI:** 10.3389/fnut.2023.1200820

**Published:** 2023-06-23

**Authors:** Guifen Wang, Meijun Ou, Hong Chen, Shujuan Zhu, Yongyi Chen, Xianghua Xu

**Affiliations:** ^1^Department of Gynecology, The Third Xiangya Hospital, Central South University, Changsha, China; ^2^Department of Nursing, The Third Xiangya Hospital, Central South University, Changsha, China; ^3^Department of Head and Neck Surgery, Hunan Cancer Hospital/The Affiliated Cancer Hospital of Xiangya School of Medicine, Central South University, Changsha, China; ^4^Hunan Cancer Hospital/The Affiliated Cancer Hospital of Xiangya School of Medicine, Central South University, Changsha, China; ^5^Health Service Center, Hunan Cancer Hospital/The Affiliated Cancer Hospital of Xiangya School of Medicine, Central South University, Changsha, China

**Keywords:** nutritional risk, oral cancer, perioperative, symptom, body mass index

## Abstract

**Introduction:**

We aimed to investigate the nutritional risk status and dynamic changes in patients with perioperative oral cancer at different stages and analyze the factors influencing nutritional risk and the correlation among body mass index, nutrition-related symptoms, and nutritional risk.

**Methods:**

In total, 198 patients with oral cancer who were hospitalized in the Head & Neck Surgery Departments of a tertiary cancer hospital in Hunan Province, China, from May 2020 to January 2021, were selected as participants. The Nutritional Risk Screening 2002 scale and Head and Neck Patient Symptom Checklist were used to assess patients on admission day, 7 days post-surgery, and 1 month post-discharge. Multivariate analysis of variance, paired *t*-test, and generalized estimating equation were used to analyze the trajectory and influencing factors of nutritional risk in patients with perioperative oral cancer. Spearman’s correlation analysis was used to explore the correlation among body mass index, symptoms, and nutritional risk.

**Results:**

The nutritional risk scores of patients with oral cancer at the three time points were 2.30 ± 0.84, 3.21 ± 0.94, and 2.11 ± 0.84, respectively, and the differences were significant (*p* < 0.05). The incidences of nutritional risk were 30.3, 52.5, and 37.9%, respectively. The factors influencing nutritional risk included education level, smoking status, clinical stage, flap repair, and tracheotomy (*β* = −0.326, 0.386, 0.387, 0.336, and 0.240, respectively, *p* < 0.05). Nutritional risk was negatively correlated with body mass index (*r_s_* = −0.455, *p* < 0.01) and positively correlated with pain, loss of appetite, sore mouth, bothersome smells, swallowing difficulty, taste changes, depression, chewing difficulty, thick saliva, and anxiety (*r_s_* = 0.252, 0.179, 0.269, 0.155, 0.252, 0.212, 0.244, 0.384, 0.260, and 0.157, respectively, *p* < 0.05).

**Conclusion:**

The incidence of nutritional risk in patients with perioperative oral cancer was high, and the trajectory of nutritional risk changed over time. Strengthening the nutritional monitoring and management of postoperative patients or those with low education level, advanced-stage cancer, flap repair, tracheotomy, and low body mass index; strengthening tobacco control management; and controlling nutrition-related discomfort symptoms in perioperative oral cancer patients are necessary.

## 1. Introduction

Oral cancer is a common type of head and neck malignancy that describes the primary malignant tumors occurring in various parts of the mouth, including the tongue, buccal mucosa, upper and lower gums, and jawbone. Nearly 377,713 new cases of oral cancer occur worldwide per year (1). The latest data released by the National Cancer Center show that there are approximately 52,200 new cases of oral cancer annually in China (2). Hunan Province has a high incidence of oral cancer. According to the latest Hunan cancer registry annual report published in 2022, the incidence of oral cancer in Hunan Province is 6.32 per 100,000, which is substantially higher than the national incidence of 3.78 per 100,000, and it continues to increase (2, 3).

Surgery is the primary treatment for oral cancer, but it can result in nutritional risks during the perioperative period (4, 5). Malignant tumors increase the body’s metabolism before surgery; however, patients may have difficulty eating owing to local pain, lumps, and ulcers in the mouth. In addition, postoperative stress causes systemic metabolic disorders, and surgical wounds can hinder patients from eating orally, necessitating feeding tubes to provide nutrition. Patients may also lack nutrition-related knowledge, guidance, and monitoring after discharge. These factors may increase the nutritional risk in patients with oral cancer during the perioperative period. A clinical study discovered that the incidence of nutritional risk in patients with oral cancer was high, at approximately 27.1% (6). Therefore, the nutritional risk of patients undergoing surgery for oral cancer requires urgent attention.

Malnutrition can increase the incidence of postoperative complications, prolong the length of hospital stay, interrupt follow-up treatment, reduce the treatment effect and quality of life of patients, and shorten the survival time of patients undergoing oral cancer surgery (7–11). Furthermore, it can decrease the body’s immune function and increase readmission rates and medical costs. The early identification of nutritional risk in patients with oral cancer at various stages during the perioperative period and understanding the factors influencing nutritional risk are essential to clarify the focus of nutritional interventions and formulate targeted nutritional intervention programs. However, current studies on the factors influencing nutritional risk in patients with oral cancer are limited by small sample sizes or incomprehensive variables, leading to inconsistent results. A study on the nutritional status and influencing factors of 50 patients with oral cancer revealed that the nutritional risk of patients with oral squamous cell carcinoma during treatment was related to education level, smoking status, and flap repair (12). Moreover, patients with advanced-stage oral cancer have a higher nutritional risk (6). The United Kingdom National Multidisciplinary Guideline on the nutritional management of head and neck cancer recommends that nutritional interventions be considered at all stages, from diagnosis to survival (13).

Most studies on the nutritional risk of patients with oral cancer are cross-sectional surveys that analyze nutritional risk from a static perspective and ignore the changing trend of nutritional risk. Research on the nutritional risk of patients with oral cancer at different stages can provide a reference and basis for clinical medical staff to adopt standardized and applicable whole-process nutritional management programs, reduce patients’ nutritional risk, and improve clinical outcomes.

Therefore, we proposed the following research hypothesis: the nutritional risk of patients with oral cancer has different characteristics and changes at different stages of surgery, and nutritional risk is affected by sociodemographic factors, disease characteristics, and nutrition-related symptoms. This study aimed to investigate the perioperative nutritional risk status and dynamic changes in patients with oral cancer and analyze the factors influencing nutritional risk.

## 2. Materials and methods

### 2.1. Study design and participants

This study utilized a longitudinal descriptive design. A literature review revealed that preoperative patients are at high nutritional risk due to various factors (6). Approximately 7 days after the operation, patients experienced a sharp decline in swallowing function, prominent nutrition-related symptoms, and poor nutritional status. However, the patients’ wounds healed, and their swallowing and other functions gradually recovered 1 month post-surgery. The severity of symptoms decreased to a level similar to that before surgery, and the nutritional status improved (14, 15). Therefore, the investigation was conducted on the day of admission (Time 1), 7 days post-surgery (Time 2), and 1 month post-discharge (Time 3) after expert consultation and group discussion. The participants were recruited from three Head & Neck Surgery Departments at a tertiary cancer hospital in Hunan Province, China, using convenience sampling between May 2020 and January 2021. The sample size was calculated as follows: $n=\frac{\mu_{\alpha /2}^{2}\pi \left(1-\pi \right)}{\delta^{2}}$ (16), where π is the population rate, the allowable error δ is 0.07, and *α* = 0.05. According to a literature review, the incidence of nutritional risk in patients with preoperative oral cancer is 32.6% (17). The sample size was calculated as 172 cases using the above equation, and the final required sample size was estimated to be 189 cases considering a 10% loss of follow-up rate and invalid samples.

The Medical Ethics Committee of the University of South China (Approval No. January 6, 2020) approved this study, and all participants provided written informed consent. The inclusion criteria were age ≥ 18 years, a diagnosis of oral cancer using pathology, preparation for surgical treatment, consciousness, and normal reading and comprehension ability. The exclusion criteria were a history of organ transplantation or malignant tumors in other body parts; the presence of gastrointestinal diseases, severe hydrothorax, ascites, or edema; and an inability to cooperate.

### 2.2. Sociodemographic and clinical characteristics

Sociodemographic data, including sex, age, job, nationality, place of residence, marital status, education level, income, medical insurance, smoking history, alcohol consumption, and betel nut chewing, were collected. Clinical data, including clinical stage, tumor location, flap repair, lymph node dissection, and tracheotomy, were also collected.

### 2.3. Body mass index

BMI was determined based on the standards of the Working Group on Obesity in China (18). It is calculated by dividing a person’s weight in kilograms by their square of height in meters. The BMI categories are defined as follows: 18.5 kg/m^2^ ≤ BMI < 24.0 kg/m^2^ is considered normal weight; BMI < 18.5 kg/m^2^ is considered underweight, indicating malnutrition; 24.0 kg/m^2^ ≤ BMI < 27.9 kg/m^2^ indicates overweight; and BMI ≥ 28.0 kg/m^2^ indicates obesity.

### 2.4. Nutritional risk

The Nutritional Risk Screening 2002 is a screening scale developed by the Working Group of the European Society of Parenteral and Enteral Nutrition through a systematic review of 128 randomized controlled studies worldwide (19). It is constructed from evidence-based medicine and has the advantages of being simple and non-traumatic. The Chinese Society of Parenteral and Enteral Nutrition recommends using this scale for nutritional risk screening in Chinese inpatients. The Nutritional Risk Screening 2002 assesses impaired nutritional status (based on unintentional weight loss, reduced food intake, and BMI) and disease severity. Each predictor is scored from 0 to 3 points, with patients aged ≥70 years receiving an extra point. The total score is the sum of these three component and ranges from 0 to 7. A total score of ≥3 points indicates that the patient has a nutritional risk.

### 2.5. Nutrition-related symptoms

The Head and Neck Patient Symptom Checklist (HNSC) was developed in 2013 by Schmidt et al. (20) to assess nutrition-related symptoms in patients with head and neck cancer. In 2019, Jin et al. (21) translated and verified the HNSC, demonstrating good reliability and validity of the Chinese version for use in clinical practice. The scale has 17 items, including 12 common and 5 systemic symptoms. Nutrition-related symptoms experienced by patients with head and neck cancer over the past 3 days were assessed using a Likert-5 scale (1 meaning “not at all,” 2 meaning “a little bit,” 3 meaning “somewhat,” 4 meaning “quite a bit,” and 5 meaning “a lot”). The sensitivity, specificity, positive predictive value, and negative predictive value of HNSC were 79–98%, 99–100%, 92–100%, and 94–100%, respectively. The Cronbach’s α coefficient of the HNSC in this study was 0.862.

### 2.6. Statistical analysis

Statistical analyses were performed using IBM SPSS Statistics for Windows (version 25.0; IBM Corp). The measurement data are described as means and standard deviations, while the count data are expressed as frequencies and constituent ratios. Multivariate analysis of repeated measurement data and paired *t*-tests were used to analyze nutritional risk status and change patterns in patients undergoing oral cancer surgery at different time points. A generalized estimating equation was used to analyze the factors influencing nutritional risk. Spearman’s correlation analysis was used to explore the correlation among BMI, symptoms, and the nutritional risk of patients with oral cancer on the day of admission. Statistical significance was set at *p* < 0.05 (two-sided).

## 3. Results

### 3.1. Characteristics of study participants

At Time 1, 218 questionnaires were distributed; all 218 were valid and collected. At Time 2, 208 valid questionnaires were collected (seven patients declined to participate, and three patients were discharged and lost to follow-up). At Time 3, 198 valid questionnaires were collected (six patients declined to participate, and four patients were lost to follow-up). Therefore, 218 questionnaires were distributed in this survey, and 198 valid questionnaires were collected, resulting in an effective response rate of 90.83%.

Table 1 presents the sociodemographic and clinical characteristics of the study participants. The average age of the participants was 52.67 years (standard deviation, 10.21; range, 30–87). Of the patient population, 172 were male (86.9%), and 26 were female (13.1%). There were 191 married (96.5%), 4 unmarried (2%), and 3 divorced/widowed people (1.5%).

**Table 1:** General information of the oral cancer surgery patients (*n* = 198).

Variables	*n*	%
Sex		
Male	172	86.9
Female	26	13.1
Age (years)		
<60	156	78.8
≥60	42	21.2
Job		
Farmers or unemployed	97	49.0
Workers	53	26.7
Staff	14	7.1
Retired	17	8.6
Self-employed	17	8.6
Nationality		
Han nationality	188	94.9
Other	10	5.1
Place of residence		
Rural area	118	59.6
Town	36	18.2
Urban area	44	22.2
Marital status		
Unmarried	4	2.0
Married	191	96.5
Divorced/widowed	3	1.5
Education level		
Elementary school or below	52	26.3
Junior middle school	104	52.5
Senior middle school	27	13.6
College or above	15	7.6
Income per month (yuan)		
<3,000	83	41.9
3,000–5,000	77	38.9
>5,000	38	19.2
Medical insurance		
Basic Medical Insurance for Urban and Rural Residents	168	84.8
Basic Medical Insurance for Employees	30	15.2
Smoking		
Yes	141	71.2
No	57	28.8
Alcohol consumption		
Yes	77	38.9
No	121	61.1
Betel nut chewing		
Yes	105	53.0
No	93	47.0
Clinical stages		
I	36	18.1
II	50	25.3
III	62	31.3
IV	50	25.3
Tumor location		
Tongue	97	50.5
Buccal mucosa	60	29.3
Gums	18	9.1
Jawbone	10	5.6
Other	13	2.5
Flap repair		
Yes	151	76.3
No	47	23.7
Lymph node dissection		
Yes	167	84.3
No	31	15.7
Tracheotomy		
Yes	46	76.8
No	152	23.2

### 3.2. Changes in nutritional risk at different time points

**Figure 1:**
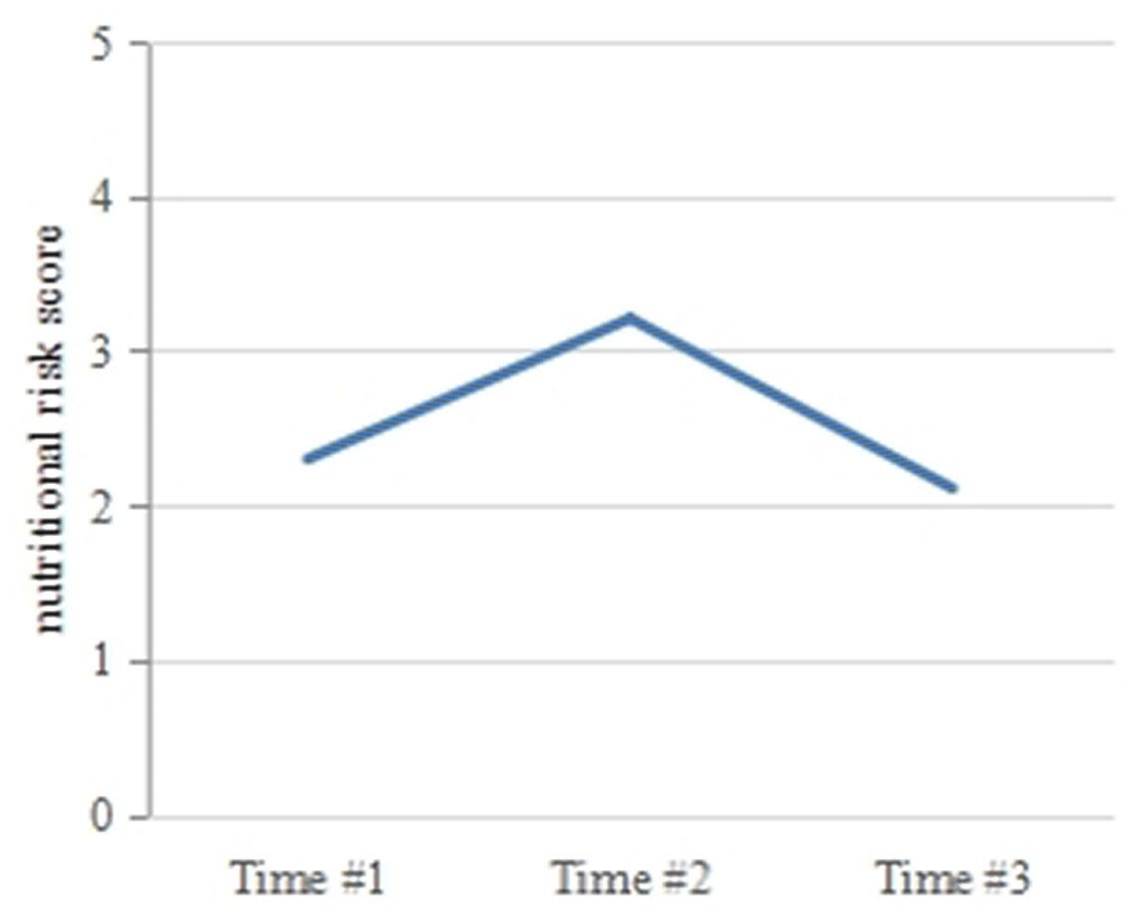
Changes in nutritional risk scores (*n* = 198).

Table 2 and Figure 1 show the changes in nutritional risk scores at the three time points. The incidence rates of nutritional risk at Times 1, 2, and 3 were 30.3, 52.5, and 37.9%, respectively. The scores of the Nutritional Risk Screening 2002 at the three time points were 2.30 ± 0.84, 3.21 ± 0.94, and 2.11 ± 0.84, respectively. Nutritional risk at the three time points was compared using multivariate analysis of repeated measurement data, and the *F* value of the Hotelling *T*^2^ test was selected. Significant differences were observed in the nutritional risk scores at the three time points (*p* < 0.05). A paired *t*-test was used to compare the nutritional risk scores at Time 2 and Time 3 with the score at Time 1, and the differences were significant (*t* = −14.521, 2.798; *p* = 0.000, 0.006). The nutritional risk score increased from Time 1 to Time 2, while the score at Time 3 decreased, with the score at Time 3 being lower than that at Time 1. The nutritional risk score at Time 2 was the highest, while that at Time 3 was the lowest.

**Table 2:** Changes in nutritional risk at three time points.

Variable	Time 1	Time 2	Time 3
	Mean (SD)	Mean (SD)	Mean (SD)
NRS 2002	2.30 (0.84)	3.21 (0.94)*	2.11 (0.84)*
*F*	939.877*		

NRS, Nutritional Risk Screening; SD, standard deviation. *P < 0.05 compared with Time 1.

### 3.3. Influencing factors of nutritional risk

A generalized estimating equation was used to longitudinally analyze the factors influencing nutritional risk. Table 3 shows the parameter estimates of the factors influencing nutritional risk. The results revealed that education level, smoking status, clinical stage, flap repair, and tracheotomy were significant factors influencing nutritional risk, with regression coefficients of −0.326, 0.386, 0.387, 0.336, and 0.240, respectively (all *p* < 0.05). Patients who graduated from senior middle school had lower nutritional risk scores than those who graduated from elementary school or below. Patients with stage IV disease, smoking history, flap repair, and tracheotomy had higher nutritional risk scores than patients with stage I disease, no smoking history, no flap repair, and no tracheotomy.

**Table 3:** Generalized estimating equation parameter estimates of factors influencing nutritional risk.

Variables	*β*	SE	95%CI		Wald χ^2^	*p-*value
			Lower bound	Upper bound		
Sex						
Male	−0.155	0.199	−0.544	0.235	0.606	0.436
Female	Reference	–	–	–	–	–
Age (years)						
≥60	0.000	0.133	−0.260	0.261	0.000	0.998
<60	Reference	–	–		–	–
Job						
Self-employed	0.029	0.134	−0.234	0.292	0.047	0.828
Retired	0.076	0.230	−0.376	0.527	0.109	0.742
Staff	0.471	0.289	−0.096	1.037	2.651	0.103
Workers	0.022	0.121	−0.216	0.260	0.031	0.859
Farmers or unemployed	Reference	–	–	–	–	–
Nationality						
Han nationality	−0.281	0.312	−0.894	0.331	0.810	0.368
Other	Reference	–	–	–	–	–
Place of residence						
Urban area	0.237	0.140	−0.039	0.512	2.839	0.092
Town	0.033	0.142	−0.245	0.312	0.055	0.815
Rural area	Reference	–	–	–	–	–
Marital status						
Divorced/widowed	−0.129	0.399	−0.912	0.653	0.105	0.746
Married	0.114	0.201	−0.279	0.507	0.322	0.571
Unmarried	Reference	–	–	–	–	–
Education level						
College or above	−0.237	0.200	−0.628	0.154	1.410	0.235
Senior middle school	−0.326	0.147	−0.615	−0.037	4.888	0.027
Junior middle school	−0.103	0.111	−0.320	0.114	0.872	0.350
Elementary school or below	Reference	–	–	–	–	–
Income per month (yuan)						
>5,000	0.096	0.126	−0.151	0.344	0.581	0.446
3,000–5,000	0.077	0.114	−0.146	0.299	0.454	0.500
<3,000	Reference	–	–	–	–	–
Medical insurance						
Basic Medical Insurance for Urban and Rural Residents	0.305	0.209	−0.104	0.715	2.135	0.144
Basic Medical Insurance for Employees	Reference	–	–	–	–	–
Smoking						
Yes	0.386	0.140	0.112	0.660	7.631	0.006
No	Reference	–	–	–	–	–
Alcohol consumption						
Yes	−0.069	0.102	−0.268	0.131	0.455	0.500
No	Reference	–	–	–	–	–
Betel nut chewing						
Yes	−0.041	0.094	−0.225	0.144	0.186	0.666
No	Reference	–	–	–	–	–
Clinical stages						
IV	0.387	0.160	0.074	0.701	5.886	0.015
III	0.252	0.155	−0.052	0.555	2.646	0.104
II	0.055	0.144	−0.228	0.338	0.147	0.702
I	Reference	–	–	–	–	–
Tumor location						
Other	−0.062	0.215	−0.484	0.360	0.084	0.773
Jawbone	0.170	0.249	−0.318	0.658	0.467	0.494
Gums	0.220	0.147	−0.068	0.509	2.241	0.134
Buccal mucosa	0.060	0.101	−0.137	0.258	0.355	0.551
Tongue	Reference	–	–	–	–	–
Flap repair						
Yes	0.336	0.135	0.072	0.600	6.231	0.013
No	Reference	–	–	–	–	–
Lymph node dissection						
Yes	−0.192	0.160	−0.505	0.122	1.433	0.231
No	Reference	–	–		–	–
Tracheotomy						
Yes	0.240	0.106	0.033	0.446	5.153	0.023
No	Reference	–	–	–	–	–

SE, standard error; CI, confidence interval.

### 3.4. Correlation among BMI, symptoms, and nutritional risk

Table 4 shows BMI, symptom scores and Spearman’s correlation analysis of BMI, symptoms, and nutritional risk at Time 1. Nutritional risk was negatively correlated with BMI (*r_s_* = −0.455, *p* < 0.01), which was positively correlated with pain, loss of appetite, sore mouth, bothersome smells, swallowing difficulty, taste changes, depression, chewing difficulty, thick saliva, and anxiety (*r_s_* = 0.252, 0.179, 0.269, 0.155, 0.252, 0.212, 0.244, 0.384, 0.260, and 0.157, respectively; *p* < 0.05).

**Table 4:** BMI, symptom scores, and correlation analysis with nutritional risk.

Variables	Score Mean (SD)	NRS 2002
BMI	23.65 (3.08)	−0.455**
HNSC		
Pain	2.37 (1.12)	0.252**
Dry mouth	1.77 (0.96)	0.053
Loss of appetite	1.39 (0.77)	0.179*
Constipation	1.39 (0.79)	0.116
Feeling full	1.31 (0.73)	−0.039
Diarrhea	1.17 (0.55)	0.019
Sore mouth	2.37 (1.09)	0.269**
Nausea	1.16 (0.56)	0.086
Vomiting	1.12 (0.49)	0.121
Bothersome smells	1.20 (0.63)	0.155*
Swallowing difficulty	1.78 (1.17)	0.252**
Taste changes	1.33 (0.75)	0.212**
Lack of energy	1.57 (0.90)	0.067
Depression	1.34 (0.72)	0.244**
Chewing difficulty	2.19 (1.21)	0.384**
Thick saliva	1.82 (1.06)	0.260**
Anxiety	1.65 (0.95)	0.157*

SD, standard deviation; NRS, Nutritional Risk Screening; BMI, body mass index; HNSC, Head and Neck Patient Symptom Checklist.

*P < 0.05.

**P < 0.01.

## 4. Discussion

This study revealed that the nutritional risk score of patients with oral cancer increased from admission to 7 days post-surgery but decreased 1 month post-discharge, indicating that the nutritional risk score fluctuates with the treatment process. The incidences of nutritional risk on the day of admission, 7 days post-surgery, and 1 month post-discharge were 30.3, 52.5, and 37.9%, respectively. The nutritional risk in patients with perioperative oral cancer is high. Yao et al. (22) conducted a study on nutritional risk screening for patients with oral and maxillofacial cancer, and the results revealed that the incidences of preoperative and postoperative nutritional risks were 27.1 and 71.2%, respectively, which were similar to our results. Postoperative nutritional risk in patients with oral cancer may be related to postoperative stress, pain, enteral nutrition, and its complications. Patients ate less than they did before surgery, resulting in decreased body weight and a significant increase in nutritional risk. As the wound healed, the patient’s food intake increased, and the body weight gradually increased compared with that on 7 days post-surgery, and the nutritional risk score decreased after discharge. However, the incidence of nutritional risk in patients after discharge remains higher than that before surgery, which could be attributed to the need for tube feeding in some patients after discharge, postoperative stress, lack of nutritional knowledge, and impaired swallowing function (23). Thus, nutritional follow-up and management of postoperative patients during the recovery period are crucial and require attention. Medical staff can strengthen nutritional support and guidance for patients with oral cancer at home through oral nutritional supplements and continuous care. At the same time, we should focus on postoperative patients, strengthen the dynamic screening and assessment of nutritional risk, identify patients with nutritional risk in time, and provide nutritional support.

Education level is an influencing factor of nutritional risk in perioperative patients with oral cancer. Our results revealed that patients who graduated from senior middle school had lower nutritional risk scores than those who graduated from elementary school or below. A previous study has also shown that patients with higher education levels have lower nutritional risk scores than those with low education levels (24). The reason may be that patients who graduated from senior middle school receive more education, possess more practical and scientific diet-related knowledge, and pay more attention to their health, while patients who graduated from elementary school or below may lack nutrition-related knowledge. Smoking is also an independent factor influencing the nutritional risk in patients with perioperative oral cancer. Gariballa et al. (25) reported that smoking causes taste decline and loss of appetite, which affects patients’ dietary intake. Compared with non-smoking patients, smokers have lower body weight, triceps skinfold thickness, and serum albumin levels, as well as worse nutritional status. Thus, smoking can increase the nutritional risk in patients with oral cancer. Medical staff should pay more attention to patients who smoke and have a low education level, provide them with nutritional guidance, educate them and their families about the hazards of smoking, and encourage smoking cessation to reduce nutritional risks and improve treatment outcomes.

Clinical stage is another influencing factor of nutritional risk in perioperative patients with oral cancer. This study’s results indicate that patients with clinical stage IV have a higher nutritional risk score than patients with clinical stage I. Patients in clinical stage IV have a long course of disease, extensive lesions, and severe clinical symptoms, leading to reduced food intake, which can create challenges in meeting the nutritional requirements of the body. The scope of surgical resection in patients with advanced-stage cancer is larger, and the damage to the physiological structures of the head and neck is greater, resulting in more severe swallowing and chewing difficulties (26, 27). After surgery, tube feeding is often the only source of nutrition, and there is a reduction in daily activities, which negatively affects patient’ BMI, muscle, and fat mass. Patients in advanced stages usually require comprehensive treatment, including radiotherapy and chemotherapy, which also increases their nutritional risk (28). This finding suggests that medical staff should pay more attention to the nutritional risk of patients with clinical stage IV.

Patients who underwent flap repair had higher nutritional risk during the perioperative period, which may be related to greater surgical trauma, and longer postoperative recovery time. Tracheotomy is another factor influencing nutritional risk scores in patients with perioperative oral cancer. Our results revealed that the nutritional risk score of patients who underwent tracheotomy was higher than that of those who did not. This may be due to the long duration of indwelling tracheotomy cannula in patients undergoing tracheotomy, which affects their comfort and can lead to complications, such as dysphagia and infection (29). Therefore, medical staff should focus on the nutritional risk of patients undergoing tracheotomy and flap repair, help patients with airway and flap management, prevent infection and other complications, and promote patient comfort to reduce their nutritional risk.

This study also showed that BMI was negatively correlated with nutritional risk and that the lower the BMI, the higher the nutritional risk score. A low BMI can reduce patients’ ability to tolerate side effects of treatment and can even lead to treatment interruption (30). Medical staff should focus on the nutritional status and risks of patients with low BMI, formulate standardized nutritional support plans for them as soon as possible, provide timely nutritional interventions to maintain appropriate weight, reduce the occurrence of nutrition-related complications, and promote their rehabilitation (30, 31).

The nutritional risk of patients on the day of admission was positively correlated with pain, loss of appetite, sore mouth, bothersome smells, swallowing difficulty, taste changes, depression, chewing difficulty, thick saliva, and anxiety, indicating that patients with more severe symptoms had higher nutritional risk scores. Pain and sore mouth were positively correlated with nutritional risk. Crowder et al. (32) reported that pain is an important factor influencing nutritional risk in patients with head and neck cancer, consistent with our results. Nutritional risk was positively associated with swallowing difficulty, which was also confirmed in the study by Morioka et al. (33). Oral malignant tumors are located in the oral cavity, and cancer cells infiltrate tissues, organs, muscles, and nerves related to swallowing, significantly affecting this function and the nutritional intake of patients (27). In addition, dysphagia increases the risk of aspiration pneumonia, resulting in increased energy expenditure, metabolic disorders, and further malnutrition (23, 34–36). Patients at nutritional risk have a higher incidence of taste changes than patients without, as also found in previous studies (37, 38). Owing to changes in taste and smell, patients cannot taste their food, affecting the pleasure and satisfaction of eating. Thick saliva was associated with nutritional risk, possibly owing to poor appetite and decreased digestive function in such patients. Chewing difficulty was also associated with nutritional risk, and Depeyre et al. found similar results (39). The invasion of cancer cells into organs involved in chewing can lead to chewing difficulty, which restricts food choices and the ability to eat a regular diet. Nutritional risk was also closely related to anxiety and depression, which is consistent with the findings of Chabowski et al. (40). Patients with cancer might experience anxiety, depression, and other adverse psychological conditions owing to worry, economic pressure, treatment-related adverse reactions, and other factors (41). Anxiety and depression can also increase caloric consumption by patients. Additionally, loss of appetite is an important factor that affects nutritional risk (42). Owing to the influence of an oral mass and these symptoms, patients may experience varying degrees of appetite loss, resulting in insufficient intake of calories, weight loss, malnutrition, and increased nutritional risk. Therefore, improving the management of preoperative pain, loss of appetite, sore mouth, bothersome smells, swallowing difficulty, taste changes, depression, chewing difficulty, thick saliva, and anxiety is crucial. Medical staff should assist patients in oral care; provide patients with adequate medication, including analgesic drugs, following the doctor’s advice; monitor the patient’s appetite and eating situation daily; and encourage patients to eat more high-calorie, high-protein, light, and easily digestible food. A quiet, comfortable, and clean dining environment should be provided as much as possible. When necessary, enteral and parenteral nutritional support should be provided (43). Patients with swallowing difficulty should undergo swallow function training as soon as possible (44). Health education, dietary guidance, and psychological counseling should be provided to patients to help them realize the importance of nutritional treatment, ensure adequate nutritional intake, and improve their nutritional status.

This study updates our knowledge of the nutritional risk in patients with perioperative oral cancer. The prospective design enabled us to survey perioperative nutritional risk over time and identify the predictors of nutritional risk in patients with oral cancer. Moreover, the analysis of influencing factors using the generalized estimating equation model allowed us to eliminate confounding factors. However, this study had several limitations. First, despite active communication measures such as telephone calls and text messages, 20 out of 218 patients were lost to follow-up owing to refusal and loss of contact. Future research should involve more active communication before surgery to reduce loss to follow-up. Second, the study only followed up patients for 1 month after discharge, which also contributed to the lack of some important clinical parameters such as mortality; hence, long-term follow-up and management are necessary for oral cancer survivors. In the future, we will continue to improve follow-up efforts and extend the follow-up time, as well as perform intervention studies based on the results of this study to confirm the significance of nutritional intervention for malnutrition and improve the nutritional status of perioperative patients with oral cancer.

## 5. Conclusion

The incidence of nutritional risk in patients with perioperative oral cancer was high, and the trajectory of nutritional risk changed over time. Strengthening the nutritional monitoring and management of postoperative patients or those with low education level, advanced-stage cancer, flap repair, tracheotomy, and low BMI; strengthening tobacco control management; and controlling nutrition-related discomfort symptoms, such as pain, loss of appetite, sore mouth, bothersome smells, swallowing difficulty, taste changes, depression, chewing difficulty, thick saliva, and anxiety, are crucial.

## Data availability statement

The original contributions presented in the study are included in the article/supplementary material, further inquiries can be directed to the corresponding author.

## Ethics statement

The Medical Ethics Committee of the University of South China (Approval No. January 6, 2020) approved this study. All participants provided written informed consent.

## Author contributions

GW, YC, and XX conceived and designed the study. GW, MO, and HC collected the data. MO and XX contributed to the data and analysis tools. GW and SZ performed the analysis. GW and XX wrote the manuscript. All authors have read and approved the final manuscript.

## Funding

This study was supported by the Health Commission of Hunan Province, China (20201091, 202214055110, and B202314027903) and the Hunan Cancer Hospital Climb Plan (QH2021004).

## Conflict of interest

The authors declare that the research was conducted in the absence of any commercial or financial relationships that could be construed as a potential conflict of interest.

## Publisher’s note

All claims expressed in this article are solely those of the authors and do not necessarily represent those of their affiliated organizations, or those of the publisher, the editors and the reviewers. Any product that may be evaluated in this article, or claim that may be made by its manufacturer, is not guaranteed or endorsed by the publisher.

## Abbreviations

HNSC: Head and Neck Patient Symptom Checklist
BMI: body mass index

## References

[1] Sung H Ferlay J Siegel RL Laversanne M Soerjomataram I Jemal A. Global Cancer Statistics 2020: GLOBOCAN estimates of incidence and mortality worldwide for 36 cancers in 185 countries. CA Cancer J Clin. (2021) 71:209–49. doi: 10.3322/caac.21660, PMID: 33538338

[2] Zheng R Zhang S Zeng H Wang S Sun K Chen R. Cancer incidence and mortality in China, 2016. J Natl Cancer Cent. (2022) 2:1–9. doi: 10.1016/j.jncc.2022.02.002 PMC11256658 39035212

[3] Xiao YZ Wang J. Hunan cancer registry annual report 2021. Changsha: Central South University Press (2022).

[4] Shanti RM O'Malley BW Jr. Surgical management of oral cancer. Dent Clin N Am. (2018) 62:77–86. doi: 10.1016/j.cden.2017.08.005, PMID: 29126495

[5] Kristensen MB Isenring E Brown B. Nutrition and swallowing therapy strategies for patients with head and neck cancer. Nutrition. (2020) 69:110548. doi: 10.1016/j.nut.2019.06.028, PMID: 31563019

[6] Gan SY. Clinical study of nutritional risk screening and nutritional support in oral cancer. Southwest medical university. Master’s Thesis. Luzhou: Southwest Medical University (2017).

[7] Wang EY Chen MK Hsieh MY Kor CT Liu YT. Relationship between preoperative nutritional status and clinical outcomes in patients with head and neck cancer. Nutrients. (2022) 14:5331. doi: 10.3390/nu14245331, PMID: 36558490 PMC9782741

[8] Findlay M White K Brown C Bauer JD. Nutritional status and skeletal muscle status in patients with head and neck cancer: impact on outcomes. J Cachexia Sarcopenia Muscle. (2021) 12:2187–98. doi: 10.1002/jcsm.12829, PMID: 34676673 PMC8718020

[9] Hiraoka SI Shimada Y Kawasaki Y Akutagawa M Tanaka S. Preoperative nutritional evaluation, surgical site infection, and prognosis in patients with oral cancer. Oral Surg Oral Med Oral Pathol Oral Radiol. (2022) 134:168–75. doi: 10.1016/j.oooo.2022.01.009, PMID: 35430178

[10] Hoang BV Tran TT Duong YT Nguyen LT Ngo DQ Nguyen DV. The effects of nutrition intervention on postoperative patients with tongue cancer and floor of mouth Cancer. J Nutr Sci Vitaminol. (2022) 68:488–95. doi: 10.3177/jnsv.68.488, PMID: 36596546

[11] Bao X Liu F Lin J Chen Q Chen L Chen F. Nutritional assessment and prognosis of oral cancer patients: a large-scale prospective study. BMC Cancer. (2020) 20:146. doi: 10.1186/s12885-020-6604-2, PMID: 32087695 PMC7036168

[12] Maierhaba M Bai J Rusitanmu Y Yao ZT. Influencing factors of nutritional status and change in 50 patients with oral squamous cell carcinoma during treatment. Shanghai J Stomatol. (2022) 31:205–10. doi: 10.19439/j.sjos.2022.02.018, PMID: 36110082

[13] Talwar B Donnelly R Skelly R Donaldson M. Nutritional management in head and neck cancer: United Kingdom National Multidisciplinary Guidelines. J Laryngol Otol. (2016) 130:S32–40. doi: 10.1017/S0022215116000402, PMID: 27841109 PMC4873913

[14] Hu ZY Feng XQ Fu MR Yu R Zhao HL. Symptom patterns, physical function and quality of life among head and neck cancer patients prior to and after surgical treatment: a prospective study. Eur J Oncol Nurs. (2020) 46:101770. doi: 10.1016/j.ejon.2020.101770, PMID: 32504879

[15] Kalavrezos N Cotrufo S Govender R Rogers P Pirgousis P Balasundram S. Factors affecting swallow outcome following treatment for advanced oral and oropharyngeal malignancies. Head Neck. (2014) 36:47–54. doi: 10.1002/hed.23262, PMID: 23559533

[16] Wang X Ji X. Sample size estimation in clinical research: from randomized controlled trials to observational studies. Chest. (2020) 158:S12–20. doi: 10.1016/j.chest.2020.03.010 32658647

[17] Li XE Lu Q Li PJ Gan L Li L. Screening of the nutritional risk of patients with oral carcinoma before operation. Chinese Nurs Manag. (2010) 10:65–7.

[18] Working Group on Obesity in China. Guidelines for prevention and control of overweight and obesity in Chinese adults (excerpt). Acta Nutri Sin. (2004):1–4.

[19] Kondrup J Allison SP Elia M Vellas B Plauth M. Educational and clinical practice committee, European Society of Parenteral and Enteral Nutrition (ESPEN). ESPEN guidelines for nutrition screening 2002. Clin Nutr. (2003) 22:415–21. doi: 10.1016/s0261-5614(03)00098-0, PMID: 12880610

[20] Schmidt KN Olson K Kubrak C Parliament M Ghosh S. Validation of the head and neck patient symptom checklist as a nutrition impact symptom assessment tool for head and neck cancer patients. Support Care Cancer. (2013) 21:27–34. doi: 10.1007/s00520-012-1483-y, PMID: 22588710

[21] Jin S Lu Q Pang D Sun Y Xiao S Zheng B. Validation of the Chinese version of the head and neck patient symptom checklist for measuring nutrition impact symptoms during radiotherapy in patients with head and neck cancer. Support Care Cancer. (2019) 27:4705–11. doi: 10.1007/s00520-019-04784-3 30949830

[22] Yao JH Zhang MF Zhang HF Ma BL Yin QM Tang W. Nutritional risk screening and enteral nutrition in patients with oral and maxillofacial cancers. Shanghai J Stomatol. (2011) 20:101–5. 21451909

[23] Sadakane-Sakuramoto A Hasegawa Y Sugahara K Horii N Saito S Nakao Y. Change in nutritional status and dysphagia after resection of head and neck Cancer. Nutrients. (2021) 13:2483. doi: 10.3390/nu13072438, PMID: 34371947 PMC8308483

[24] Song C Cao J Zhang F Wang C Guo Z Lin Y. Nutritional risk assessment by scored patient-generated subjective global assessment associated with demographic characteristics in 23,904 common malignant tumors patients. Nutr Cancer. (2019) 71:50–60. doi: 10.1080/01635581.2019.1566478, PMID: 30741002

[25] Gariballa S Forster S. Effects of smoking on nutrition status and response to dietary supplements during acute illness. Nutr Clin Pract. (2009) 24:84–90. doi: 10.1177/0884533608329441, PMID: 19244153

[26] Kim D Li R. Contemporary treatment of locally advanced oral cancer. Curr Treat Options in Oncol. (2019) 20:32. doi: 10.1007/s11864-019-0631-8, PMID: 30874958

[27] Hasegawa T Yatagai N Furukawa T Wakui E Saito I Takeda D. The prospective evaluation and risk factors of dysphagia after surgery in patients with oral cancer. J Otolaryngol Head Neck Surg. (2021) 50:4. doi: 10.1186/s40463-020-00479-6, PMID: 33494830 PMC7830751

[28] Shibahara T. Oral cancer -diagnosis and therapy. Clin Calcium. (2017) 27:1427–33. 28947694

[29] Reddy PD Yan F Nguyen SA Nathan CO. Factors influencing the development of pneumonia in patients with head and neck cancer: a meta-analysis. Otolaryngol Head Neck Surg. (2021) 164:234–43. doi: 10.1177/0194599820938011, PMID: 32660345

[30] Ackerman D Laszlo M Provisor A Yu A. Nutrition management for the head and neck cancer patient. Cancer Treat Res. (2018) 174:187–208. doi: 10.1007/978-3-319-65421-8_11 29435843

[31] Sandmael JA Sand K Bye A Solheim TS Oldervoll L Helvik AS. Nutritional experiences in head and neck cancer patients. Eur J Cancer Care. (2019) 28:e13168. doi: 10.1111/ecc.13168 31571296

[32] Crowder SL Douglas KG Yanina Pepino M Sarma KP Arthur AE. Nutrition impact symptoms and associated outcomes in post-chemoradiotherapy head and neck cancer survivors: a systematic review. J Cancer Surviv. (2018) 12:479–94. doi: 10.1007/s11764-018-0687-7, PMID: 29556926

[33] Morioka R Matsuda Y Kato A Okui T Okuma S Tatsumi H. Oral functional impairment may cause malnutrition following oral cancer treatment in a single-center cross-sectional study. Sci Rep. (2022) 12:14787. doi: 10.1038/s41598-022-19177-6, PMID: 36042270 PMC9428164

[34] Gallegos C Brito-de la Fuente E Clavé P Costa A Assegehegn G. Nutritional aspects of dysphagia management. Adv Food Nutr Res. (2017) 81:271–318. doi: 10.1016/bs.afnr.2016.11.008 28317607

[35] Omura T Matsuyama M Nishioka S Sagawa S Seto M Naoe M. Association between the swallowing reflex and the incidence of aspiration pneumonia in patients with dysphagia admitted to long-term care wards: a prospective cohort study of 60 days. Arch Phys Med Rehabil. (2021) 102:2165–71. doi: 10.1016/j.apmr.2021.06.012, PMID: 34252394

[36] Sato S Takahashi H. Assessment of the risk of malnutrition due to aspiration pneumonia and oral feeding difficulty. Nutr Hosp. (2020) 37:723–9. doi: 10.20960/nh.03109, PMID: 32720506

[37] McGettigan N Dhuibhir PU Barrett M Sui J Balding L Higgins S. Subjective and objective assessment of taste and smell sensation in advanced cancer. Am J Hosp Palliat Care. (2019) 36:688–96. doi: 10.1177/1049909119832836 30827119

[38] Jin S Lu Q Sun Y Xiao S Zheng B Pang D. Nutrition impact symptoms and weight loss in head and neck cancer during radiotherapy: a longitudinal study. BMJ Support Palliat Care. (2021) 11:17–24. doi: 10.1136/bmjspcare-2019-002077, PMID: 32019753

[39] Depeyre A Pereira B Pham-Dang N Barthélémy I Hennequin M. Impairments in food oral processing in patients treated for tongue cancer. Dysphagia. (2020) 35:494–502. doi: 10.1007/s00455-019-10054-5, PMID: 31598793

[40] Chabowski M Polański J Jankowska-Polańska B Janczak D Rosińczuk J. Is nutritional status associated with the level of anxiety, depression and pain in patients with lung cancer. J Thorac Dis. (2018) 10:2303–10. doi: 10.21037/jtd.2018.03.108 29850135 PMC5949507

[41] Yuan L Pan B Wang W Wang L Zhang X Gao Y. Prevalence and predictors of anxiety and depressive symptoms among patients diagnosed with oral cancer in China: a cross-sectional study. BMC Psychiatry. (2020) 20:394. doi: 10.1186/s12888-020-02796-6, PMID: 32758185 PMC7405439

[42] Viana ECRM Oliveira IDS Rechinelli AB Marques IL Souza VF Spexoto MCB. Malnutrition and nutrition impact symptoms (NIS) in surgical patients with cancer. PLoS One. (2020) 15:e0241305. doi: 10.1371/journal.pone.0241305, PMID: 33320857 PMC7737886

[43] Ham OK Chee W Im EO. The influence of social structure on cancer pain and quality of life. West J Nurs Res. (2017) 39:1547–66. doi: 10.1177/0193945916672663, PMID: 27703078 PMC9307873

[44] Tseng WH Li TH Chiu HL Yang TL Wang CP Chen TC. Long-term swallowing-related outcomes in oral cancer patients receiving proactive swallowing therapy. Oral Oncol. (2021) 122:105569. doi: 10.1016/j.oraloncology.2021.105569, PMID: 34656054

